# Electroacupuncture alleviates cognitive impairments in APP/PS1 mice via gastric vagal afferent-mediated activation of the nucleus tractus solitarius‒locus coeruleus noradrenergic circuit

**DOI:** 10.1186/s13020-025-01230-x

**Published:** 2025-11-13

**Authors:** Chaochao Yu, Man Li, Feng Shen, Shan Gao, Jia Wang, Chuan He, Li Wang, Yongchao Wu, Yanjun Du

**Affiliations:** 1 Department of Rehabilitation, Union Hospital, Tongji Medical College, Huazhong University of Science and Technology, Wuhan, Hubei Province China; 2 School of Basic Medicine, Hubei Key Laboratory of Drug Target Research and Pharmacodynamic Evaluation, Tongji Medical College, Huazhong University of Science and Technology, Wuhan, Hubei Province China; 3 College of Acupuncture and Orthopedics, Hubei University of Chinese Medicine, Wuhan, Hubei Province China; 4 Department of Acupuncture and Moxibustion, Wuhan Hospital of Integrated Traditional Chinese and Western Medicine, Wuhan, Hubei Province China

**Keywords:** Gastrointestinal vagal afferent fiber, Nucleus tractus solitaries, Locus coeruleus, Alzheimer's disease, Neuroinflammation, Electroacupuncture

## Abstract

**Objective:**

Neuroinflammatory cascades mediated by the locus coeruleus-norepinephrine (LC-NE) system emerge as critical pathophysiological determinants governing the etiology and advancement of neurodegenerative disorders, including Alzheimer's disease (AD). Therapeutic modulation of neuroinflammatory processes may mitigate AD-associated cognitive decline. Electroacupuncture (EA) targeting the Foot Yangming of Stomach Meridian acupoints demonstrates efficacy in ameliorating AD-related cognitive dysfunction through dual peripheral and central anti-inflammatory actions, though the precise neural circuitry linking peripheral interventions to central effects remains undefined.

**Methods:**

In this study, APP/PS1 transgenic mice were administrated with EA stimulation at ST36 and ST37. Cognitive function was assessed via the Morris water maze and novel object recognition tests. Spatial learning capacities and episodic memory retention were quantified through Morris water maze paradigm and object novelty discrimination assays, respectively. Retrograde neural tracing was employed to validate the nucleus tractus solitarius (NTS)-LC noradrenergic projection. Multimodal approaches integrating chemogenetic manipulation, immunofluorescence microscopy, Western blotting, Golgi-Cox neuronal morphology analysis, and Nissl histochemistry elucidated the gastrointestinal vagal afferent fiber (GVAF)-NTS-LC circuit's role in EA-mediated neuroinflammatory regulation. Circuit-specific functional validation was conducted through selective GVAF blockade.

**Results:**

EA at ST36 and ST37 attenuated hippocampal synaptic ultrastructural degeneration via NTS-LC noradrenergic circuit activation. This intervention suppressed proinflammatory cytokines expression (IL-6, IL-1β, TNF-α) and microglial hyperactivation through ADRB2/PKA/CREB pathway modulation, effectively rescuing cognitive deficits in AD models. GVAF ablation reversed EA-induced neuroinflammatory suppression, confirming the circuit dependence.

**Conclusions:**

EA at ST36/ST37 alleviates AD-related cognitive impairment through sequential GVAF-NTS-LC circuit activation and downstream ADRB2/PKA/CREB-mediated neuroinflammatory resolution. This work identifies a previously unrecognized peripheral-central neural circuit mechanism underlying EA's therapeutic efficacy in AD pathogenesis.

## Introduction

Alzheimer's disease (AD) is the most prevalent progressive and debilitating neurodegenerative disorder and is characterized by gradual memory decline and cognitive dysfunction [1, 2]. Its primary pathological hallmarks include extracellular amyloid deposits forming senile plaques and intracellular neurofibrillary tangles composed of the hyperphosphorylated microtubule-associated protein Tau [1–3]. These pathological changes are often accompanied by synaptic dysfunction [3, 4], glial cell activation [3, 5], and neuronal degeneration and loss [3, 6]. According to the 2023 World Alzheimer's Disease Report, approximately 55 million people worldwide were living with AD in 2023, with the number increasing at a rate of three individuals per second. It is projected that this figure will rise to 139 million by 2050 [7]. As global aging intensifies, the development of potential strategies for the treatment of AD has become an urgent challenge for international public health.

Multiple pathways ascending from the gastrointestinal tract to the brain, such as the gut microbiota pathway, neural signaling pathway, and neuroimmune interaction pathway, can transmit gastrointestinal physiological and pathological information to the brain, thereby participating in the regulation of brain functional states [8–10]. Peripheral vagal afferent fibers project to the nucleus tractus solitarius (NTS) in the brainstem, which further extends to several critical nuclei, including the locus coeruleus (LC), dorsal raphe nucleus, hypothalamus, and basal ganglia, thereby modulating physiological activities such as memory, emotion, feeding, and immune responses [11]. Studies have revealed that the gastrointestinal vagal afferent fiber (GVAF)-medial septum-hippocampus circuit is involved in regulating spatial and episodic memory in mice. The molecular mechanism involves GVAF-mediated excitatory modulation of glutamatergic neurons within the CA3 and dentate gyrus, as well as the upregulation of brain-derived neurotrophic factor (BDNF) expression [12]. In contrast, mice subjected to subdiaphragmatic vagotomy exhibit reduced hippocampal neurogenesis and decreased BDNF expression levels [13]. Clinical studies have demonstrated that vagus nerve stimulation therapy can enhance memory performance in elderly individuals [14, 15] and improve cognitive function in patients with AD [16] and vascular dementia [17]. Therefore, vagus nerve stimulation therapy may represent a promising approach for ameliorating cognitive impairment in AD patients.

Contemporary clinical research incorporating multicenter RCTs and systematic meta-analyses has established the therapeutic efficacy of acupuncture in mitigating Alzheimer's disease-related cognitive decline [18]. Zusanli (ST36) and Shangjuxu (ST37), belonging to the Stomach Meridian of Foot-Yangming, are the “Xiahe points” of the stomach and large intestine according to the meridian theory, respectively. They share homologous innervation and play pivotal roles in regulating gut-brain co-morbid disorders [19–22]. Electroacupuncture (EA) at ST36 improves cognitive function in AD, with mechanisms involving the regulation of gut microbiota homeostasis [23], alleviation of cerebral microvascular damage and hypoperfusion [24], and enhancement of long-term potentiation (LTP) [25] in various AD animals, including 3xTg-AD mice, APP/PS1 mice, and D-galactose-induced AD-like pathology rats. Studies indicate that EA at ST36 exerts systemic anti-inflammatory effects by activating vagal‒adrenal anti-inflammatory axis [26] and can suppress neuroinflammation in AD by inhibiting microglial activation [27]. Our prior experimental findings established that EA at ST36 mitigates hippocampal neuroinflammation and rescues cognitive dysfunction in AD-like pathology rats through the modulation of intestinal microbial homeostasis and suppression of TLR4/NF-κB signaling [23]. It has been proved that acupuncture at ST36-ST37 attenuated hippocampal inflammation in epileptic seizure rats [28]. Additionally, acupuncture at ST37 showed neuroprotective effects in Parkinson’s disease mice [29] and cerebral ischemic stroke mice [30], indicating that ST37 may also serve as a vital acupoints in treating nervous system diseases, including AD [31]. Somatosensory stimulation via acupuncture has been established to mediate homeostatic regulation through bidirectional modulation of neuro-autonomic pathways, particularly involving parasympathetic vagal afferents and adrenergic neural circuitry [32–35]. However, the mechanistic basis through which EA modulates neuroinflammatory cascades in Alzheimer's pathology via vagal-brain axis regulation and associated neural circuitry reorganization warrants rigorous investigation. In this study, we demonstrated that EA at ST36 and ST37 alleviates neuroinflammation and cognitive deficits in APP/PS1 double transgenic mice via gastric vagal afferent-mediated activation of the NTS ‒ LC noradrenergic circuit.

## Material and methods

### Animals

The Ethics Committee of Hubei University of Chinese Medicine approved all experimental protocols (Approval ID: HUCMS13243481). All the mice were 6 months old and weighed 25.0–30 g. The present study utilized male APP/PS1 mice as the AD model, with C57BL/6 J mice serving as controls. The animals were kept in separate cages under controlled environmental conditions, including a 12-h light/dark cycle, a stable temperature range of 20–22 °C, and a relative humidity of 50% ± 10%. Before the study commenced, the mice were acclimatized for at least 7 days and were provided with free access to food and water. Group allocation was performed via a random number method.

### Retrograde monosynaptic transsynaptic viral tracing

APP/PS1 mice aged 6 months were anesthetized and subjected to stereotaxic surgery under conditions consistent with prior methodologies [36]. To achieve retrograde trans-monosynaptic tracing of upstream NTS inputs to noradrenergic neurons in the LC, a combination of three helper adeno-associated viruses (AAVs) and a modified rabies virus was employed as follows: AAV9-DIO-TVA-GFP; AAV9-DIO-RVG; AAV9-TH-Cre (serotype 9 AAV encoding Cre recombinase under the tyrosine hydroxylase promoter for targeted expression in noradrenergic neurons); and RV-ENVA-ΔG-dsRed (EnvA-pseudotyped, glycoprotein-deleted rabies virus expressing dsRed fluorophore).

A mixture of the three helper viruses (AAV9-DIO-TVA-GFP, AAV9-DIO-RVG, and AAV9-TH-Cre) was prepared at a 1:1:1 volumetric ratio (total injection volume: 300 nL) and stereotaxically microinjected into the LC (from bregma: anterior–posterior (AP) + 0.3 mm, mediolateral (ML) − 1.0 mm, and dorsal–ventral (DV) − 4.05 mm). The mice were allowed to recover for 3 weeks to ensure robust Cre-mediated recombination and stable expression of the TVA receptor, rabies glycoprotein (RG), and GFP in TH-positive LC neurons. The modified rabies virus RV-ENVA-ΔG-dsRed (total injection volume: 120 nL) was administered into the same LC coordinates.

After 10 days of rabies virus propagation and retrograde transsynaptic labeling, the mice were transcardially perfused with fixative. The brains were extracted, cryosectioned, and imaged via laser scanning confocal microscopy to visualize GFP-labeled TH neurons and dsRed-labeled presynaptic input neurons.

### Chemogenetic viral injection

The procedures for chemogenetic viral injection, mouse anesthesia, and stereotaxic surgery were the same as those described previously [36]. We employed chemogenetic techniques to achieve bidirectional modulation of the NTS-LC noradrenergic circuit. Using stereotaxic methods, we microinjected recombinant adeno-associated viral (rAAV) vectors into the LC of mice for noradrenergic neuron-specific manipulation. Experimental groups received 200 nl of viral solution containing hM3D(Gq), hM4D(Gi), or mCherry reporter genes (injection rate: 40 nl/min), co-administered with TH promoter-driven Cre recombinase virus (rAAV-TH-Cre) to enable noradrenergic neuron-specific expression. To eliminate neural circuit compensatory effects, the EA + hM4D group received bilateral LC injections, while other groups underwent unilateral left-sided injections. Following a 3-week viral transfection period (ensuring stable receptor protein expression), clozapine N-oxide (CNO, 1 mg/kg) or equivalent saline was administered intraperitoneally 30 min before EA intervention for chemogenetic activation/inhibition. The hM3D group received CNO to enhance neuronal activity, while the EA + hM4D group received CNO to suppress activity. mCherry control groups concurrently received equivalent saline or CNO to account for nonspecific drug effects.

### Gastrointestinal vagal afferent fiber blockade

The specific blockade of gastrointestinal vagal afferent fibers was performed via the cholecystokinin-saporin (CCK-SAP) injection method into the nodose ganglion, as described by Charlene Diepenbroek et al. [37] This approach offers a significant advantage over traditional subdiaphragmatic vagotomy, as CCK-SAP selectively binds to CCK receptors on vagal afferent neurons, thereby blocking the transmission of gastrointestinal-derived vagal afferent signals while preserving efferent vagal signaling. A reduction in food intake by 30% or more compared with the preoperative level was considered indicative of successful blockade [12].

### EA treatment

Three weeks postviral injection, the mice were subjected to EA treatment. The EA protocol was adapted from our previous study [36]. The animals were gently restrained in the supine position via custom-designed clothing restraints without the use of anesthesia. The mice received EA stimulation at acupoints ST36 and alternating unilateral ST37. ST36 is located on the anterior aspect of the hindlimb, approximately 2 mm below the knee joint, whereas ST37 is situated on the lateral side of the hindlimb, approximately 5 mm below the knee joint, as per the standardized mouse acupoint atlas [38]. Stainless steel needles (13 mm in length × 0.25 mm in diameter) were inserted bilaterally at acupoints ST36 and ST37 under standardized protocols. For ST36 positioning, needles were inserted at a 15° oblique angle to achieve a tissue depth of 3–5 mm, whereas vertical insertion (90°) was employed at ST37 with equivalent penetration depth. All penetrated needles were interfaced with an electroacupuncture stimulator (HANS-100A, China), programmed to deliver continuous electrical pulses at 2 Hz frequency and 2 mA amplitude with the pulse width at 300 μs. The treatment lasted 30 min and was administered every other day for 1 month. Throughout the procedure, the animals remained calm and exhibited no signs of distress. The mice administrated with sham EA intervention underwent the same restraint but were only subjected to shallow subcutaneous needling without connecting to the nerve stimulator device.

### Novel object recognition (NOR) test

The NOR paradigm was employed to investigate the effects of EA on declarative memory consolidation in the mice over a 3-day period. Initially, the mice underwent 10-min exposure to a neutral arena (40 × 40 × 50 cm^3^) devoid of sensory stimuli to establish spatial familiarity on the first day. The following day, they were placed back into the same setup, which now contained two similar objects, A and B. The NOR test included two phases, a 10-min acquisition phase and a 10-min familiarization phase, which were conducted on the third day. During the acquisition phase, objects A and B were presented again. In the familiarization phase, object B was replaced with a perceptually distinct novel object C which was unfamiliar to the mice. Exploration was defined as nasal/whisker contact ≤ 2 cm from object surface and, excluding sitting or walking near the objects. The recognition index (RI) was derived by normalizing the temporal allocation toward novel stimulus investigation against the cumulative exploration duration of both objects [39].

### Morris water maze (MWM) test

The MWM test was performed to examine the spatial learning and memory capabilities of the mice according to the protocol outlined in our previous study [36]. The behavioral testing system comprised a cylindrical pool (100 cm diameter × 50 cm elevation) maintained in a thermoregulated environment (24 °C ± 2 °C). This aquatic maze was partitioned into four geometrically congruent sectors, with an alabaster escape platform (10 cm diameter) positioned 1 cm subaquatically from the liquid interface. Over a 5-day period, the mice were given one minute daily to find the hidden platform. On the sixth day, a probe trial was conducted to evaluate memory retention. A ceiling-mounted video acquisition system continuously captured locomotor trajectories in the WMW paradigm, with subsequent computational processing quantifying platform acquisition latency and temporal allocation within the target quadrant.

### Golgi staining

The Golgi impregnation technique was carried out via a commercial Golgi staining system (Servicebio, Wuhan, China) under ambient temperature conditions. Initially, cerebral tissues were immersed in a composite solution containing reagents A and B for a 2-week fixation period. The samples were subsequently transferred to reagent C for secondary processing for 2–7 days. Tissue sectioning was performed via a Leica CM1860S microtome to obtain 60 µm thick slices, which were then mounted onto glass slides. Following two sequential 4-min rinses with ddH₂O, the prepared slides were finally incubated in a ternary mixture comprising reagent C, reagent D, and deionized water. The dendritic spine density per 10 μm was quantified via ImageJ software.

### Nissl's staining

After dehydrating the tissue samples through a series of graded alcohol solutions (75%, 85%, 90%, and 95%), they were sliced using a cryostat. The sections were then stained with prewarmed toluidine blue Nissl solution for 20–40 min, followed by differentiation in an alcohol gradient (2 min per concentration) and a brief rinse in distilled water for 30 s. The sections were subsequently mounted with neutral resin and examined under a light microscope to assess the pathological morphology of the hippocampal neurons.

### Western blot analysis

The protein levels of ADRB2, PKA, phosphorylated PKA (p-PKA), CREB, phosphorylated CREB (p-CREB), IL-6, IL-1β, and TNF-α were measured through Western blot analysis. Hippocampal specimens were immediately excised under cryogenic dissection protocols. Protein homogenates normalized for equal loading were subjected to electrophoretic separation using sodium dodecyl sulfate–polyacrylamide gel (SDS-PAGE) systems, followed by transmembranous transfer onto PVDF matrices employing capillary-based aqueous phase methodology. The membranes were pre-treated with 5% skimmed milk powder in Tris-buffered saline (TBS-T) for 60 min under ambient conditions (24 ± 1 °C), prior to extended incubation (16 h, 4 °C) with the primary antibodies listed in Table 1. Subsequent immunodetection employed HRP-linked species-matched IgG conjugates (1 h, 25 °C), followed by chemiluminescent substrate-mediated visualization of immunoreactive bands using a gel documentation system (Cat# abs920; Abisin, China). The band intensities were analyzed via ImageJ software to quantify gray values.

**Table 1:** Antibody information

Antibodies	IF	WB	Companies	Species	Cat No
tublin	/	1:3000	Proteintech	Mouse	66200-1-Ig
IL-6	/	1:1000	ABclonal	Rabbit	A0286
IL-1β	/	1:3000	ABclonal	Rabbit	A22257
TNF-α	/	1:1500	ABclonal	Rabbit	A11534
IBA-1	1:500	/	Servicebio	Rabbit	GB153502-100
TH	1:500	/	Servicebio	Mouse	GB12181-100
DBH	1:2000	/	Abcam	Rabbit	Ab209487
Noradrenaline	1:100	/	Abcam	Rabbit	Ab254361
c-Fos	1:1000	/	Abcam	Mouse	Ab208942
ADRB2	/	1:600	ABclonal	Rabbit	A2048
PKA	/	1:500	ABclonal	Rabbit	A0798
p-PKA	/	1:500	ABclonal	Rabbit	AP0557
CREB	/	1:500	ABclonal	Rabbit	A10826
p-CREB	/	1:300	ABclonal	Rabbit	AP0903
CCK-AR	1:100	/	Santa Cruz	Mouse	Sc-514303

### Immunofluorescence staining

The mice were induced into a surgical plane of anesthesia using sodium pentobarbital (1%) and underwent transcardial perfusion with physiological saline followed by ice-cold 4% paraformaldehyde dissolved in 0.1 M phosphate-buffered saline (pH 7.4). Post-perfusion, cerebral tissues were meticulously dissected and immersion-fixed in fresh 4% PFA solution at 4 °C for 24 h. Following graded ethanol dehydration, specimens were paraffin-embedded and sectioned coronally (5 μm thickness) to encompass nuclei of interest (NTS, LC, hippocampus). These sections underwent standardized epitope unmasking procedures as previously described [36]. Histological specimens were subjected to sequential immunolabeling protocols: primary non-specific epitope masking with 10% goat serum supplemented with 0.3% Triton X-100 (37 °C, 60 min), followed by extended antigen–antibody conjugation (18 h, 4 °C) with primary immunoreagents detailed in Table 1. Subsequent immunolabeling employed species-matched fluorescent IgG conjugates (37 °C, 1 h). Processed sections were mounted and imaged using a high-resolution virtual microscopy system (VS-120, Olympus, Japan). Quantitative analysis involved systematic random sampling of cerebral sections from ≥ 4 biological replicates, with neuronal positivity determined through standardized counting protocols.

### Statistical analysis

The normality of the data was assessed via the Kolmogorov‒Smirnov test. Data conforming to a normal distribution are expressed as the means ± SDs. Statistical analyses were conducted via GraphPad Prism 8.0 software (San Diego, USA). Comparisons among groups were performed via one-way ANOVA, followed by Dunnett’s post hoc test for multiple comparisons. A p value of less than 0.05 was defined as statistically significant.

## Results

### EA effectively alleviates learning and memory deficits in APP/PS1 mice

**Fig. 1:**
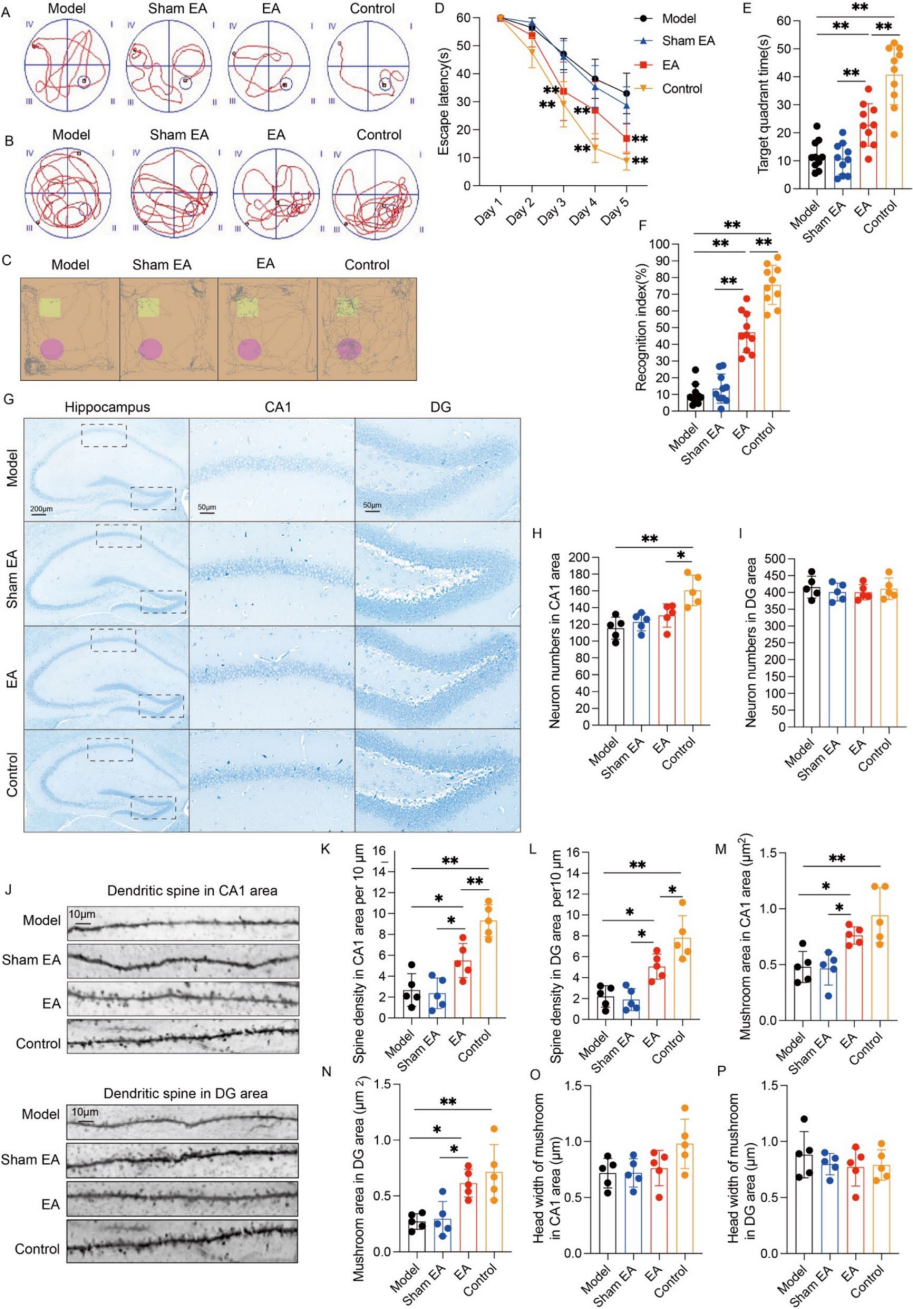
Effects of EA on learning and memory abilities and neuronal injury in APP/PS1 mice. **A** Representative spatial navigation profiles depicting mice locomotor paths during the spatial acquisition phase. **B** Representative cognitive mapping trajectories from the probe test. **C** Representative movement patterns of the mice in the novel object cognition assay. **D** Quantification of escape latency in the navigation test (mean ± SD, n = 10). **P < 0.01 by one-way ANOVA. **E** Quantification of target quadrant dwell time in the probe test (mean ± SD, n = 10). **P < 0.01 by one-way ANOVA. **F** Quantification of the recognition index in the novel object recognition test (mean ± SD, n = 10). *P < 0.05 by one-way ANOVA. **G** Representative Nissl staining showing the number of neurons in the hippocampus of the mice in each group. Scale bar: 200 μm (minification) and 50 μm (magnification). **H** Data represent the mean ± SD (n = 5). *P < 0.05 and **P < 0.01 by one-way ANOVA. **I** Data represent the mean ± SD (n = 5). *P* > 0.05 by one-way ANOVA. **J** Representative Golgi staining images showing the dendritic spine density in the hippocampal CA1 and DG areas of the mice in each group. Scale bar: 10 μm. **K**–**N** Quantitative results are presented as means ± SD (n = 5). *P < 0.05 and **P < 0.01 by one-way ANOVA. **O**, **P** Data represent the mean ± SD (n = 5). *P* > 0.05 by one-way ANOVA

In Experiment 1, the mice underwent randomized allocation into four experimental groups: non-treated controls, model, EA-treated (ST36/ST37stimulation), and sham-stimulated (non-acupoint) groups, to systematically evaluate electroacupuncture's neuromodulatory effects on Alzheimer's-associated cognitive dysfunction. Following 30-day intervention cycles, spatial learning and recognition memory were assessed via the MWM and NOR behavior tests. The MWM test revealed that the escape latency in both model and EA groups significantly exceeded control group baselines from trial day 3 onward (p < 0.01, Fig. 1D). Conversely, EA-treated mice demonstrated accelerated platform acquisition latencies compared to model subjects and sham-EA subjects, with temporal improvements emerging from experimental day 3 onward (p < 0.01, Fig. 1D). In the spatial probe test, mice in the model and EA groups spent significantly less time in the target quadrant than did those in the control group (p < 0.01, Fig. 1E). Conversely, the mice in the EA group presented prolonged target quadrant dwell times relative to those in the model and sham EA groups (p < 0.01, Fig. 1E), with no inter-group distinction between the model and sham EA groups (p > 0.05, Fig. 1E). The NOR test indicated markedly diminished RI in model and EA groups relative to controls (p < 0.01, Fig. 1F). However, EA-treated mice demonstrated significant cognitive discrimination index enhancement compared to model and sham groups (p < 0.01, Fig. 1F). Quantitative stereological analysis revealed marked depletion of hippocampal CA1 neuronal populations in the model group compared to the control group (p < 0.01, Fig. 1G, H). No intergroup variations were observed among the model, sham EA, and EA groups (p > 0.05, Fig. 1H), nor within dentate gyrus (DG) across all groups (p > 0.05, Fig. 1G, I), implying dissociation between EA-induced mnemonic enhancement and neuronal density alterations. Ultrastructural analysis demonstrated significant reductions in neuron dendritic spine density in CA1 and DG areas within the model group versus the control group (p < 0.01, Fig. 1J–L). EA treatment substantially increased spine density relative to model and sham EA groups (p < 0.05, Fig. 1J–L). EA also resulted in an increase in the size of mushroom spines in CA1 area compared to the model and sham EA groups (p < 0.05, Fig. 1M). Similar effects on the dendritic spine morphology were observed in DG area (p < 0.05, Fig. 1N). While, no significant changes in the head width of mushroom spines were observed among groups in CA1 and DG areas (p < 0.05, Fig. 1O, P). These findings suggested potential synaptotrophic effects underlying EA-mediated cognitive rescue through synaptic ultrastructural preservation.

### EA inhibits hippocampal neuroinflammation in APP/PS1 mice

**Fig. 2:**
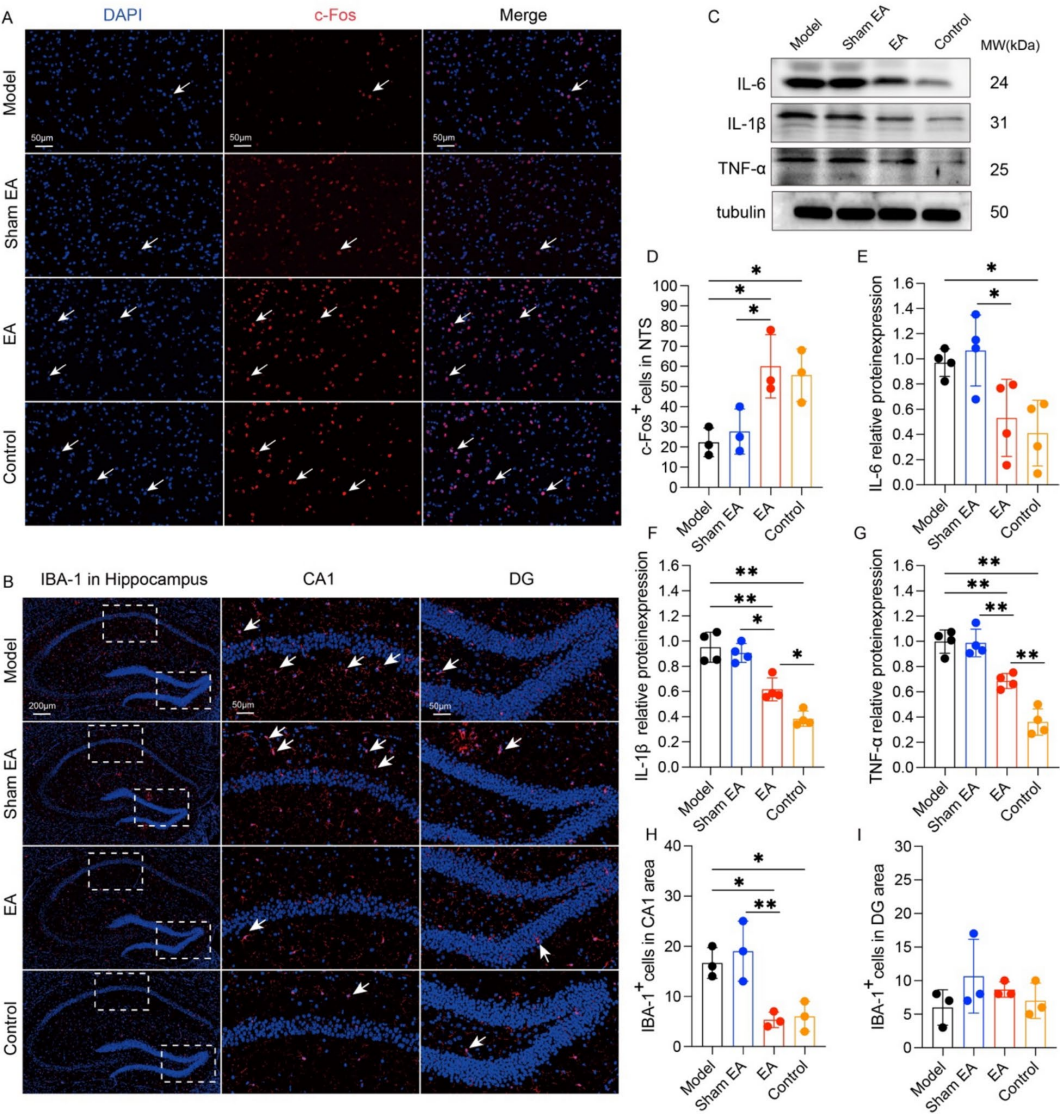
Effects of EA on hippocampal neuroinflammation in APP/PS1 mice. **A** Representative immunofluorescence images of c-Fos (red) and nuclei (blue) and their colocalization in the NTSs of the mice from each group. Scale bar: 50 μm. **B** Representative immunofluorescence images of IBA-1 (red) and nuclei (blue) and their colocalization in the hippocampus of mice from each group. Scale bar: 200 μm (minification) and 50 μm (magnification). **C** Representative Western blots showing the levels of IL-6, IL-1β, and TNF-α in the hippocampi of the mice in each group. **D** Data represent the mean ± SD (n = 3). *P < 0.05 by one-way ANOVA. **E** Data represent the mean ± SD (n = 3). *P < 0.05 by one-way ANOVA. **F** Data represent the mean ± SD (n = 4).*P < 0.05 and **P < 0.01 by one-way ANOVA. **G** Data represent the mean ± SD (n = 4). **P < 0.01 by one-way ANOVA. **H** Data represent the mean ± SD (n = 3). *P < 0.05 and **P < 0.01 by one-way ANOVA. **I** Data represent the mean ± SD (n = 3). P > 0.05 by one-way ANOVA

The brainstem NTS can respond to peripheral acupuncture stimulation and relay acupuncture signals to other brain regions [40, 41]. To investigate the potential involvement of NTS neuronal excitability in EA-mediated neurocognitive preservation, we examined c-Fos expression in the NTS following EA at ST36 and ST37. Comparative analysis revealed diminished c-Fos immunoreactive neuron density within the NTS of the model group relative to the control group (p < 0.05, Fig. 2A, D). EA treatment increased the number of c-Fos-positive neurons in the NTS compared with those in the model and sham EA groups (p < 0.05, Fig. 2D), indicating potential mediation of EA-induced cognitive rescue in AD through NTS neuronal hyperexcitability modulation. Neuroinflammation impairs synaptic plasticity and exacerbates AD-related cognitive deficits. Microglia play pivotal roles in AD-associated neuroinflammatory cascades. Activated microglia polarize into proinflammatory M1 phenotypes (releasing IL-1β, TNF-α, IL-6, and COX-2) and into anti-inflammatory M2 phenotypes [42, 43]. EA significantly reduced hippocampal IL-1β (p < 0.05, Fig. 2C, F) and TNF-α (p < 0.05, Fig. 2C, G) levels compared with those in the model group but did not alter IL-6 expression (p > 0.05, Fig. 2C, E). Compared with the sham EA group, the EA group presented significantly lower IL-6, IL-1β, and TNF-α expression (p > 0.05, Fig. 2C, E–G). EA markedly decreased the number of activated microglia in the hippocampal CA1 region compared with the model and sham EA groups (p < 0.01, Fig. 2B, H). No intergroup differences were detected in the DG (p > 0.05, Fig. 2B, I).

### EA modulates learning and memory in APP/PS1 mice via vagal afferent neurons

**Fig. 3:**
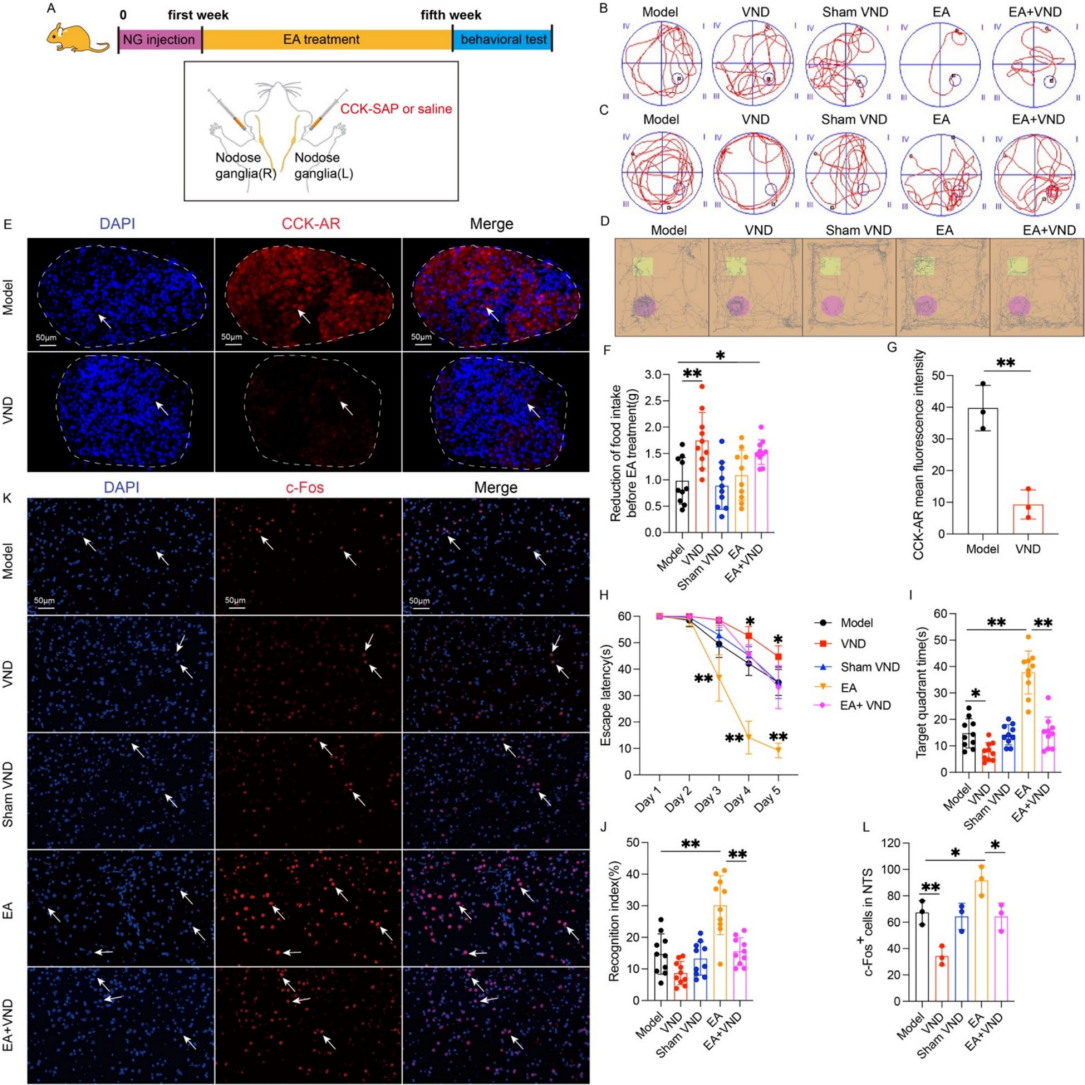
EA modulates learning and memory in APP/PS1 mice via vagal afferent neurons. **A** Workflow of the vagal afferent neuron blockade strategy and EA treatment. **B** Spatial navigation profiles depicting mice locomotor paths during the spatial acquisition phase. **C** Representative cognitive mapping trajectories from the probe test. **D** Representative movement patterns of the mice in in the novel object cognition assay. **E** Representative multiplexed fluorescence images of CCK-AR (red) and nuclei (blue) and their colocalization in the nodose ganglia of mice from model and VND groups. Scale bar: 50 μm. **F** Quantification of the reduction in food intake of the mice in each group (mean ± SD, n = 10). *p < 0.05, **p < 0.01, analyzed by one-way ANOVA. **G** Data represent the mean ± SD (n = 3). **P < 0.01 by one-way ANOVA. **H** Quantification of escape latency in the navigation test (mean ± SD, n = 10). *P < 0.05 and **P < 0.01 by one-way ANOVA. **I** Quantification of the target quadrant dwell time in the probe test (mean ± SD, n = 10). *P < 0.05 and **P < 0.01 by one-way ANOVA. **J** Quantification of the recognition index in the novel object recognition test (mean ± SD, n = 10). **P < 0.01 by one-way ANOVA. **K** Multiplexed fluorescence micrographs demonstrating c-Fos immunoreactivity (red signal), DAPI-counterstained nuclei (blue signal), and their cellular co-expression within NTS microdomains across experimental cohorts. Scale bar: 50 μm. **L** Data represent the mean ± SD (n = 3). *P < 0.05 and **P < 0.01 by one-way ANOVA

A previous study demonstrated that low-intensity EA at ST36 exerts anti-inflammatory effects by activating the vagus nerve-adrenal anti-inflammatory axis [26]. Vagal afferent fibers exhibit anatomical and functional connectivity with the LC [44, 45]. To investigate whether the EA-induced suppression of hippocampal neuroinflammation in APP/PS1 mice involves vagal afferent neurons and LC neurons, bilateral injection of CCK-SAP into the nodose ganglia (Fig. 3A) selectively damaged CCKAR-expressing vagal afferent neurons. The mice were randomly assigned to five groups, namely, the model group, VND group (vagal afferent neuron deletion), sham VND group (saline injection into the nodose ganglion), EA group, and EA + VND group. The results demonstrated that vagal nerve damage induced substantial suppression of food intake relative to the model group (p < 0.01, Fig. 3F). Furthermore, CCK-AR expression on neurons of the nodose ganglion in CCK-SAP-treated animals was significantly reduced compared with that in model group (p < 0.01, Fig. 3E, G). These results validated effective disruption of the neural pathway [12, 44]. The MWM experiments revealed significantly prolonged escape latencies in VND group from day 3 onward (p < 0.05, Fig. 3B, H), coupled with diminished target quadrant occupancy durations (p < 0.05, Fig. 3C, I). EA effectively reversed these spatial memory deficits (p < 0.01, Fig. 3C, I). NOR test showed comparable discrimination indices between VND and model groups, while EA-mediated cognitive enhancement was abrogated by vagal denervation (p < 0.01, Fig. 3D, J).

**Fig. 4:**
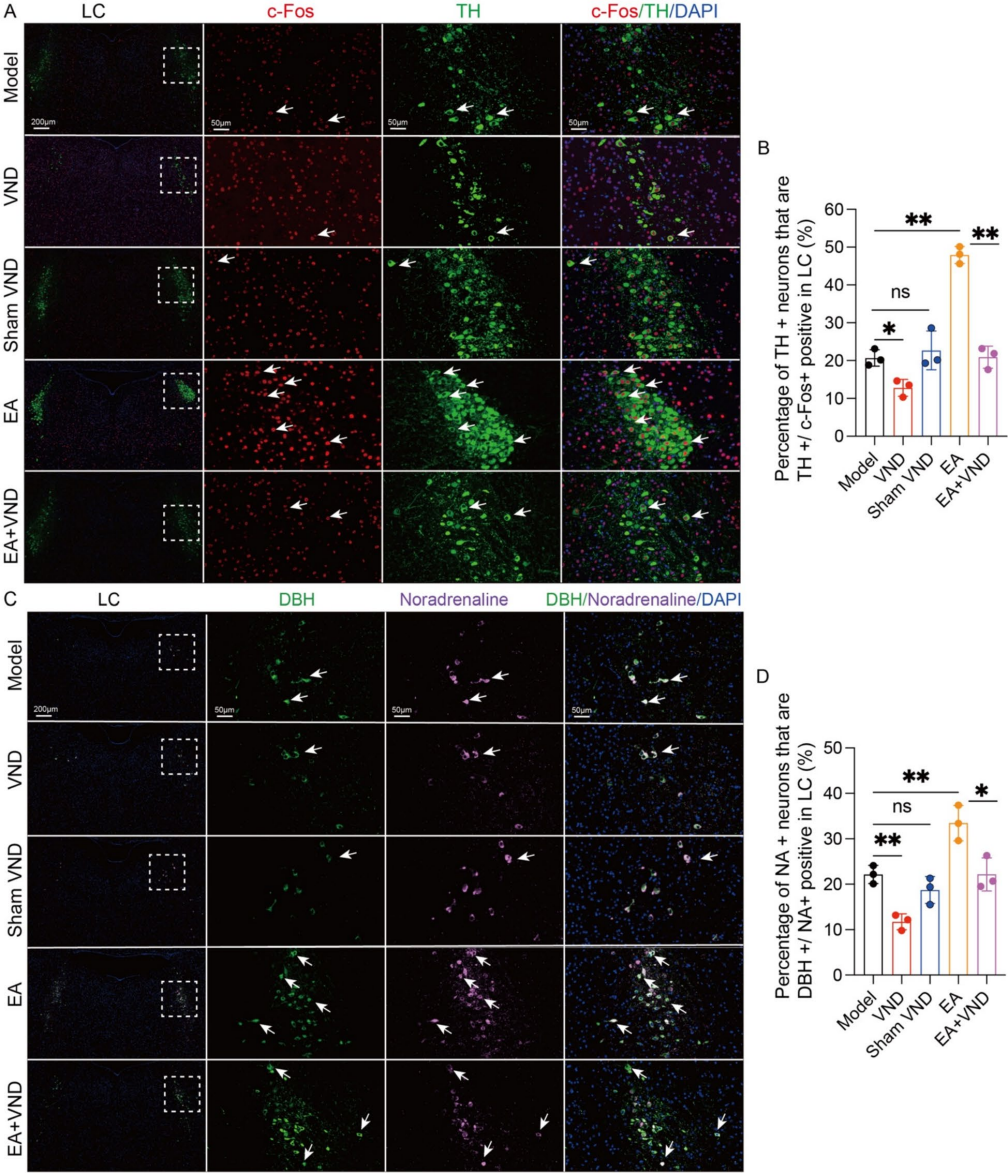
Effects of EA on the excitability of noradrenergic neurons in the LC in APP/PS1 mice after vagal afferent neuron blockade. **A** Representative multiplexed fluorescence images of c-Fos (red), TH (green), and nuclei (blue) and their colocalization in the LC of mice from each group. Scale bar: 200 μm (minification) and 50 μm (magnification). **B** Data represent the mean ± SD (n = 3). *P < 0.05 and **P < 0.01 by one-way ANOVA. **C** Representative immunofluorescence images of noradrenaline (purple), DBH (green), and nuclei (blue) and their colocalization in the LC of mice from each group. Scale bar: 200 μm (minification) and 50 μm (magnification). **D** Data represent the mean ± SD (n = 3). *P < 0.05 and **P < 0.01 by one-way ANOVA

Fluorescent immunohistochemical analysis demonstrated diminished c-Fos⁺ neuronal density in the NTS of vagotomized mice compared to the model subjects relative to disease model controls (p < 0.01, Fig. 3K, L). EA intervention upregulated c-Fos immunoreactivity (p < 0.05, Fig. 3K, L), whereas EA + VND co-treatment attenuated this activation versus EA monotherapy (p < 0.05, Fig. 3K, L), confirming EA-induced NTS neuronal excitation requires preserved vagal afferent integrity. Parallel analyses of LC neurochemistry revealed depleted percentage of TH⁺/c-Fos⁺ positive neurons in VND group (p < 0.05, Fig. 4A, B), while EA significantly amplified this neurochemical signature (p < 0.01, Fig. 4A, B). Notably, EA + VND combinatorial intervention reduced percentage of TH⁺/c-Fos⁺ positive neurons compared to EA-treated counterparts (p < 0.01, Fig. 4A, B). Complementary neurochemical profiling identified compromised NA biosynthesis in LC from VND mice (p < 0.01, Fig. 4C, D), contrasting with EA-mediated augmentation of increased percentage of DBH⁺/NA⁺ positive neurons (p < 0.01, Fig. 4C, D). As hypothesized, EA + VND co-administration reduced percentage of DBH⁺/NA⁺ positive neurons compared to EA intervention alone (p < 0.05, Fig. 4C, D). These data mechanistically link EA-dependent LC noradrenergic potentiation to vagal afferent-mediated signaling, ultimately driving cognitive remediation in APP/PS1 mice.

### Validation of NTS-LC noradrenergic projection

**Fig. 5:**
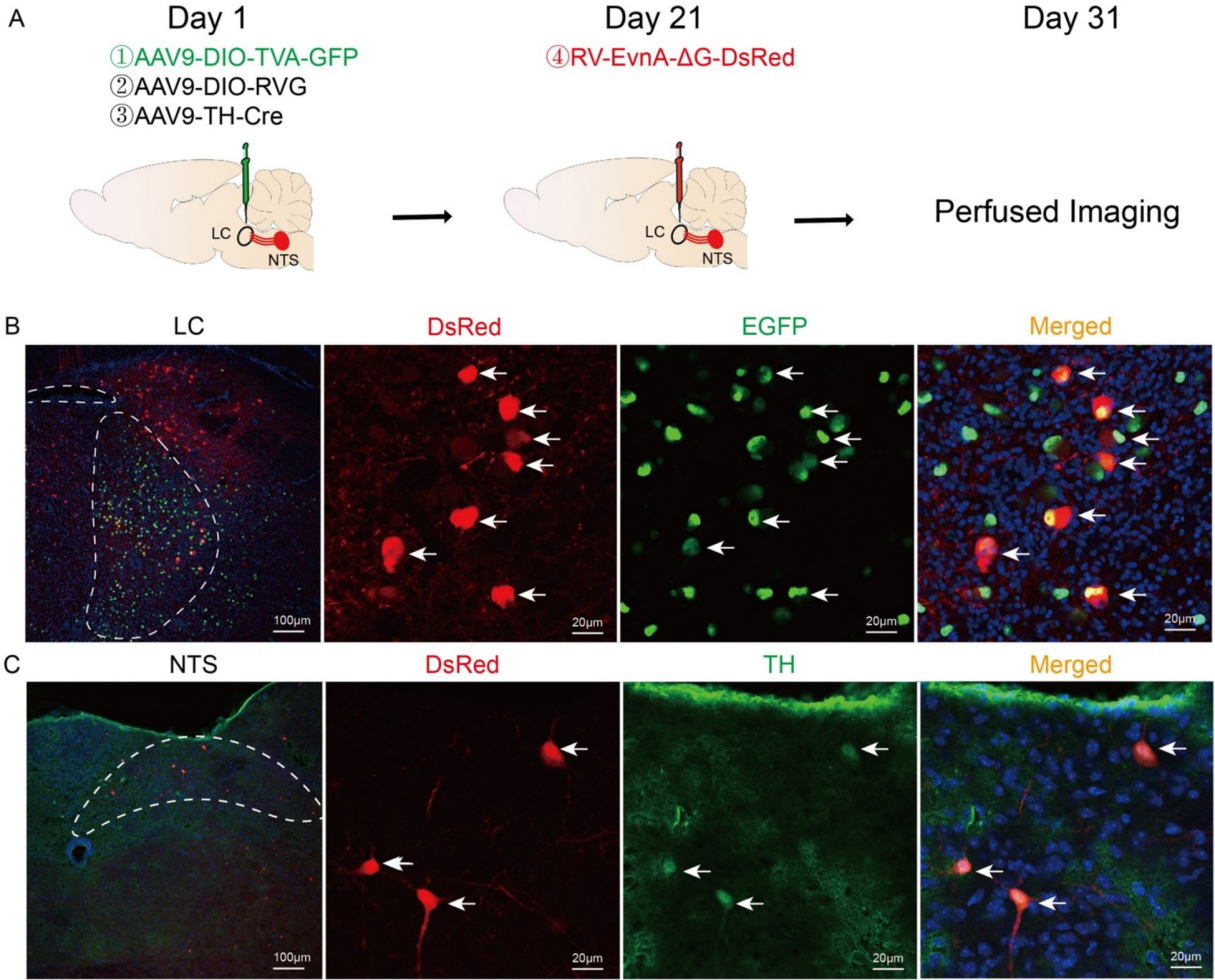
Validation of the NTS-LC noradrenergic projection. **A** Workflow of virus injection. **B** Representative multiplexed fluorescence images of DsRed (red), EGFP (green), and nuclei (blue) and their colocalization in the LC of mice from each group. Scale bar: 100 μm (minification) and 20 μm (magnification). **C** Representative multiplexed fluorescence images of DsRed (red), TH (green), and nuclei (blue) and their colocalization in the NTSs of the mice from each group. Scale bar: 100 μm (minification) and 20 μm (magnification)

Prior experimental evidence established electroacupuncture's capacity to activate TH⁺ neurons within the NTS and LC. Furthermore, vagal sensory inputs were shown to modulate LC noradrenergic neuronal excitability and NA biosynthesis. To delineate the functional connectivity of this NTS-LC noradrenergic pathway, we implemented a monosynaptic retrograde tracing strategy. Triple recombinant adeno-associated viral vectors (rAAV9-DIO-TVA-EGFP, rAAV9-DIO-RVG, rAAV9-TH-Cre) were delivered stereotactically into LC nuclei. Post 3-week viral incubation, RV-ENVA-ΔG-dsRed was microinfused into NTS. Terminal transcardial perfusion was performed after 7 days, followed by cryosectioning of coronally oriented brain specimens (Fig. 5A). High-resolution confocal imaging revealed intense EGFP expression within LC neuronal populations (Fig. 5B), with substantial overlap between dsRed⁺ neurons and TH⁺ cellular profiles in NTS (Fig. 5C). This neuroanatomical mapping conclusively verifies TH⁺ neuron-restricted dsRed expression in NTS, thereby demonstrating the existence of a monosynaptic noradrenergic efferent pathway from NTS to LC.

### The NTS-LC noradrenergic circuit mediates the EA-induced improvement in learning and memory in APP/PS1 mice

**Fig. 6:**
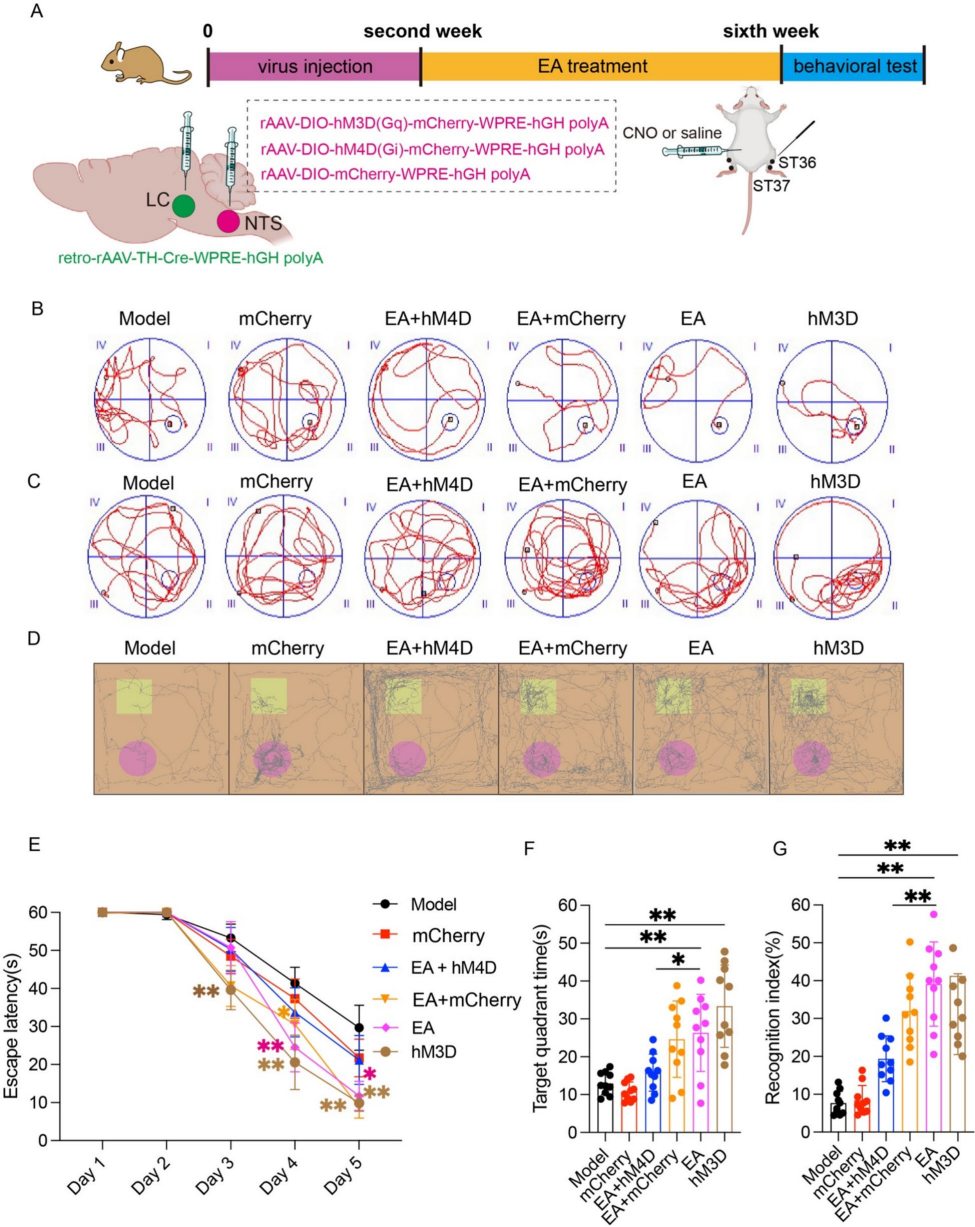
NTS-LC noradrenergic circuit mediates the EA-induced improvement in learning and memory in APP/PS1 mice. **A** Workflow of virus injection and EA treatment. **B** Spatial navigation profiles depicting mice locomotor paths during the spatial acquisition phase. **C** Representative cognitive mapping trajectories from the probe test. **D** Representative movement patterns of the mice in the novel object cognition assay. **E** Quantification of escape latency in the navigation test (mean ± SD, n = 10). *P < 0.05 and **P < 0.01 by one-way ANOVA. **F** Quantification of target quadrant dwell time in the probe test (mean ± SD, n = 10). *P < 0.05 and **P < 0.01 by one-way ANOVA. **G** Quantification of the recognition index in the NOR test (mean ± SD, n = 10). *P < 0.05 by one-way ANOVA

The prior research established that EA enhances cognitive performance in APP/PS1 transgenic mice, concurrent with noradrenergic neuronal hyperactivation within NTS and LC. Anatomical tracing further confirmed the existence of noradrenergic efferent projections from NTS to LC. The causal involvement of this NTS‒LC noradrenergic circuit in mediating EA-induced neuroprotection was validated through the chemogenetic interrogation (Fig. 6A). Complementary behavioral profiling demonstrated accelerated platform acquisition latencies in both EA-treated and hM3D-activated groups compared to the model group from experimental day 3 (p < 0.05, Fig. 6E), coupled with extended quadrant occupancy durations (p < 0.05, Fig. 6F). These findings suggest EA recapitulates hM3D-mediated NTS‒LC circuit potentiation to alleviate cognitive dysfunction. Pharmacogenetic suppression via EA + hM4D co-intervention significantly attenuated spatial memory retention compared to EA (p < 0.01, Fig. 6F), demonstrating circuit-dependent therapeutic reversal. The NOR test revealed enhanced RI in EA and hM3D groups versus model counterparts (p < 0.01, Fig. 6G), whereas hM4D co-administration abolished EA-mediated cognitive preservation (p < 0.01, Fig. 6G). This bidirectional modulation confirms NTS‒LC noradrenergic circuitry as the critical mediator of EA-induced neurocognitive benefits.

### The NTS‒LC noradrenergic circuit mediates EA-induced improvements in synaptic ultrastructural damage and neuroinflammation in APP/PS1 mice

**Fig. 7:**
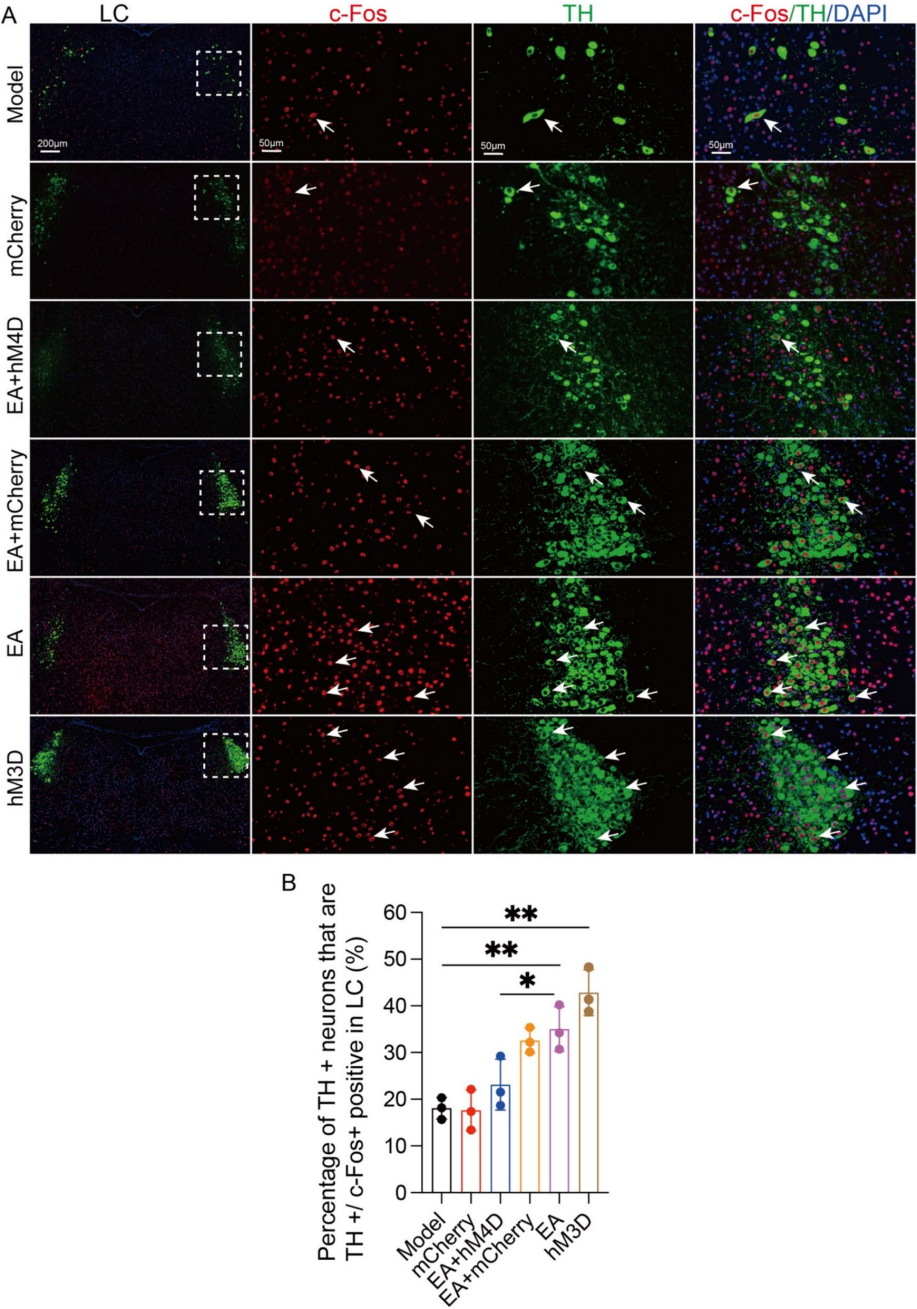
NTS-LC noradrenergic circuit mediates the EA-induced improvement in learning and memory in APP/PS1 mice. **A** Representative immunofluorescence images of c-Fos (red), TH (green), and nuclei (blue) and their colocalization in the LC of mice from each group. Scale bar: 200 μm (minification) and 50 μm (magnification). **B** Data represent the mean ± SD (n = 3). *P < 0.05 and **P < 0.01 by one-way ANOVA

**Fig. 8:**
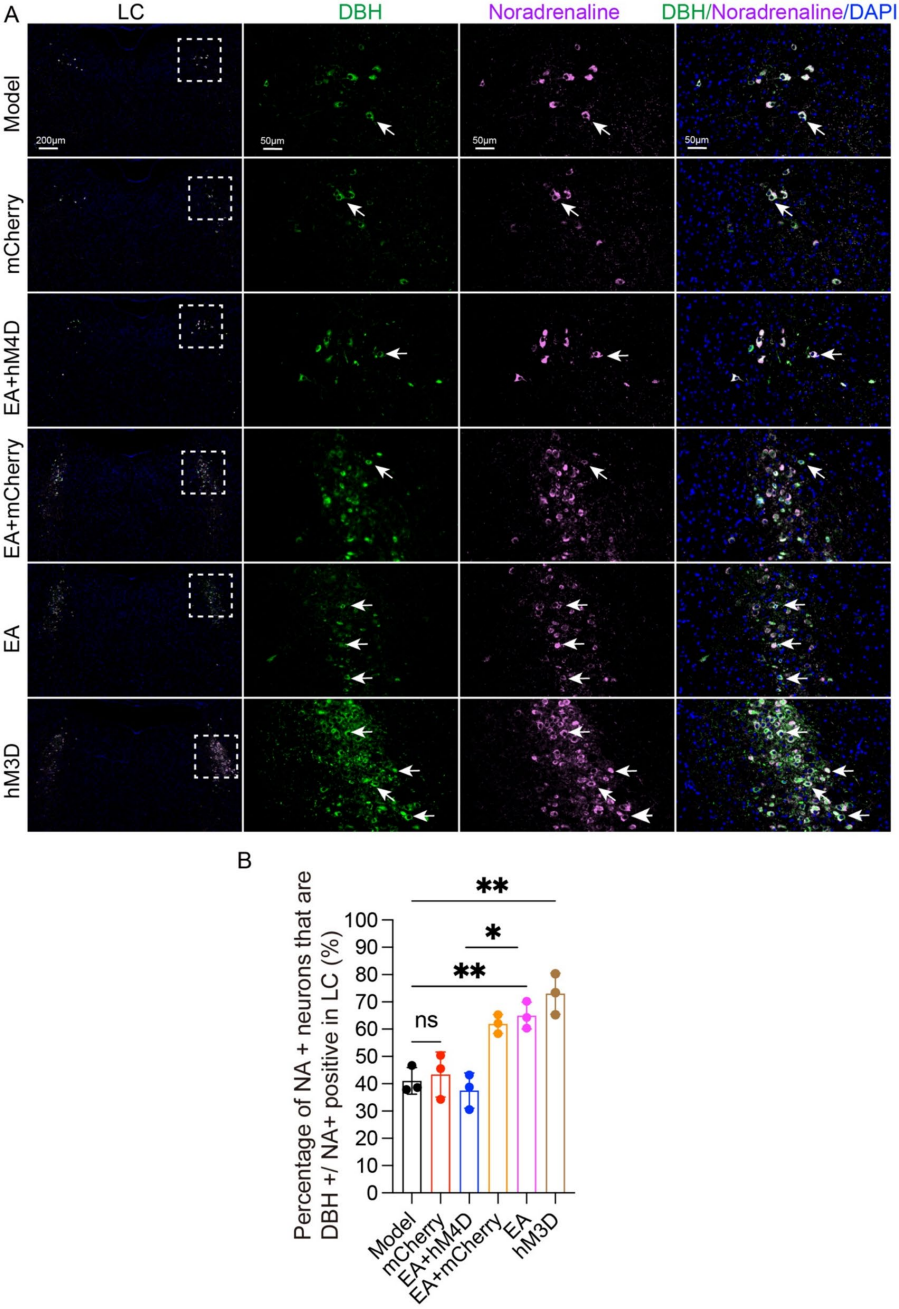
Effects of EA on noradrenaline synthesis in the LC of APP/PS1 mice. **A** Representative immunofluorescence images of noradrenaline (purple), DBH (green), and nuclei (blue) and their colocalization in the LC of mice from each group. Scale bar: 200 μm (minification) and 50 μm (magnification). **B** Data represent the mean ± SD (n = 3). *P < 0.05 and **P < 0.01 by one-way ANOVA

We subsequently evaluated the neuromodulatory effects of EA on LC noradrenergic neuronal dynamics in APP/PS1 transgenic models. Immunofluorescence co-localization analysis demonstrated marked elevation of percentage of TH-positive/c-Fos-activated (TH^+^/c-Fos^+^) neuronal density within the LC of EA-treated and hM3D-activated groups compared to the model group (p < 0.01, Fig. 7B). Pharmacogenetic inhibition via EA + hM4D co-intervention substantially attenuated TH^+^/c-Fos^+^ neuronal activation indices relative to EA (p < 0.05, Fig. 7B), confirming reversible suppression of EA-induced LC noradrenergic activation. Complementary neurochemical analysis revealed enhanced NA biosynthesis in LC from EA and hM3D groups versus the model group (p < 0.01, Fig. 8B). Strikingly, EA + hM4D combinatorial treatment significantly downregulated NA production compared to EA intervention (p < 0.05, Fig. 8B). These data collectively delineate a regulatory mechanism whereby EA potentiates LC noradrenergic neurotransmission through targeted activation of the NTS-LC circuit, thereby amplifying central NA bioavailability.

**Fig. 9:**
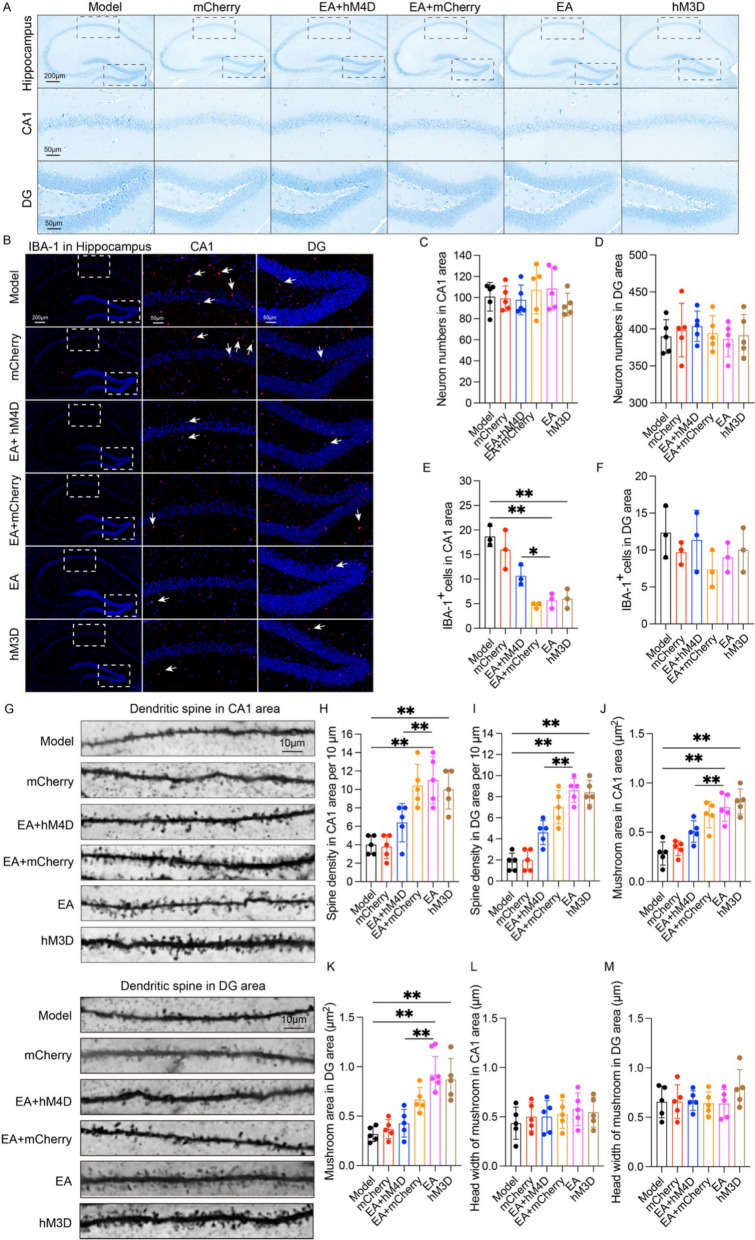
The NTS‒LC Noradrenergic Circuit Mediates EA-Induced Improvements in Synaptic Ultrastructural Damage and Neuroinflammation in APP/PS1 Mice. **A** Representative Nissl staining images showing the number of neurons in the hippocampus of the mice in each group. Scale bar: 200 μm (minification) and 50 μm (magnification). **B** Representative immunofluorescence images of IBA-1 (red) and nuclei (blue) and their colocalization in the hippocampus of mice from each group. Scale bar: 200 μm (minification) and 50 μm (magnification). **C** Data represent the mean ± SD (n = 5). *P* > 0.05 by one-way ANOVA. **D** Data represent the mean ± SD (n = 5). *P* > 0.05 by one-way ANOVA. **E** Data represent the mean ± SD (n = 3). *P < 0.05 and **P < 0.01 by one-way ANOVA. **F** Data represent the mean ± SD (n = 3). *P* > 0.05 by one-way ANOVA. **G** Representative Golgi staining images showing the dendritic spine density in the hippocampal CA1 and DG areas of the mice in each group. Scale bar: 10 μm. **H**–**K** Data represent the mean ± SD (n = 5). *P < 0.01 by one-way ANOVA. **L**, **M** Data represent the mean ± SD (n = 5). *P* > 0.05 by one-way ANOVA

We further investigated the neurobiological consequences of inhibition of the NTS-LC noradrenergic circuit on hippocampal activation, neuronal integrity, and synaptic plasticity. Nissl histochemical analysis demonstrated conserved neuronal population density across experimental groups (p > 0.05, Fig. 9A, C, D), indicating NTS-LC circuit modulation-independent neuronal survival. Neuroinflammatory profiling identified significant microglial deactivation in CA1 subareas of EA and hM3D groups relative to the model group (p < 0.01, Fig. 9B, E). Conversely, EA + hM4D combinatorial treatment amplified microglial reactivity beyond EA intervention baselines (p < 0.05, Fig. 9B, E), while microglial dynamics in DG remained intercohort invariant (p > 0.05, Fig. 9B, F). Quantitative synaptic ultrastructure assessment via Golgi-Cox impregnation revealed enhanced dendritic spine density in EA-treated and hM3D-activated groups relative to the model group (p < 0.01, Fig. 9G–I). Notably, hM4D co-intervention substantially attenuated spine density restoration achieved through EA (p < 0.01, Fig. 9G–I), demonstrating pharmacogenetic blockade of EA-mediated synaptogenesis. Besides, quantitative analysis of dendritic spines in CA1 and DG areas indicated that EA resulted in an increase in the area of mushroom spines (p < 0.05, Fig. 9J, K), but the head width of mushroom spines remained unchanged (p < 0.05, Fig. 9L, M). These findings establish a mechanistic paradigm wherein EA-mediated NTS-LC noradrenergic circuit activation exerts dual neuroprotective effects: suppression of neuroinflammatory gliosis and structural reinforcement of synaptic connectivity in APP/PS1.

### EA suppresses neuroinflammation in the hippocampus of APP/PS1 mice by activating the β2-AR/PKA/CREB pathway

Our previous findings demonstrated that EA improves cognitive deficits in APP/PS1 mice by increasing LC noradrenaline synthesis via activation of the NTS-LC noradrenergic circuit. The β2-adrenergic receptor (β2-AR)/protein kinase A (PKA)/cAMP response element-binding protein (CREB) signaling pathway has been shown to be closely associated with neuroinflammatory responses, neuroregeneration, synaptic plasticity, and memory formation [46]. β2-AR activation by NA or β2-AR agonists can trigger the cAMP/PKA/CREB pathway in microglia, thereby suppressing microglial activation and alleviating neuroinflammation [47, 48]. To further investigate the underlying anti-neuroinflammatory mechanisms of EA, we measured hippocampal protein levels of β2-AR, phosphorylated PKA (p-PKA), phosphorylated CREB (p-CREB), and inflammatory cytokines (IL-6, IL-1β, and TNF-α).

**Fig. 10:**
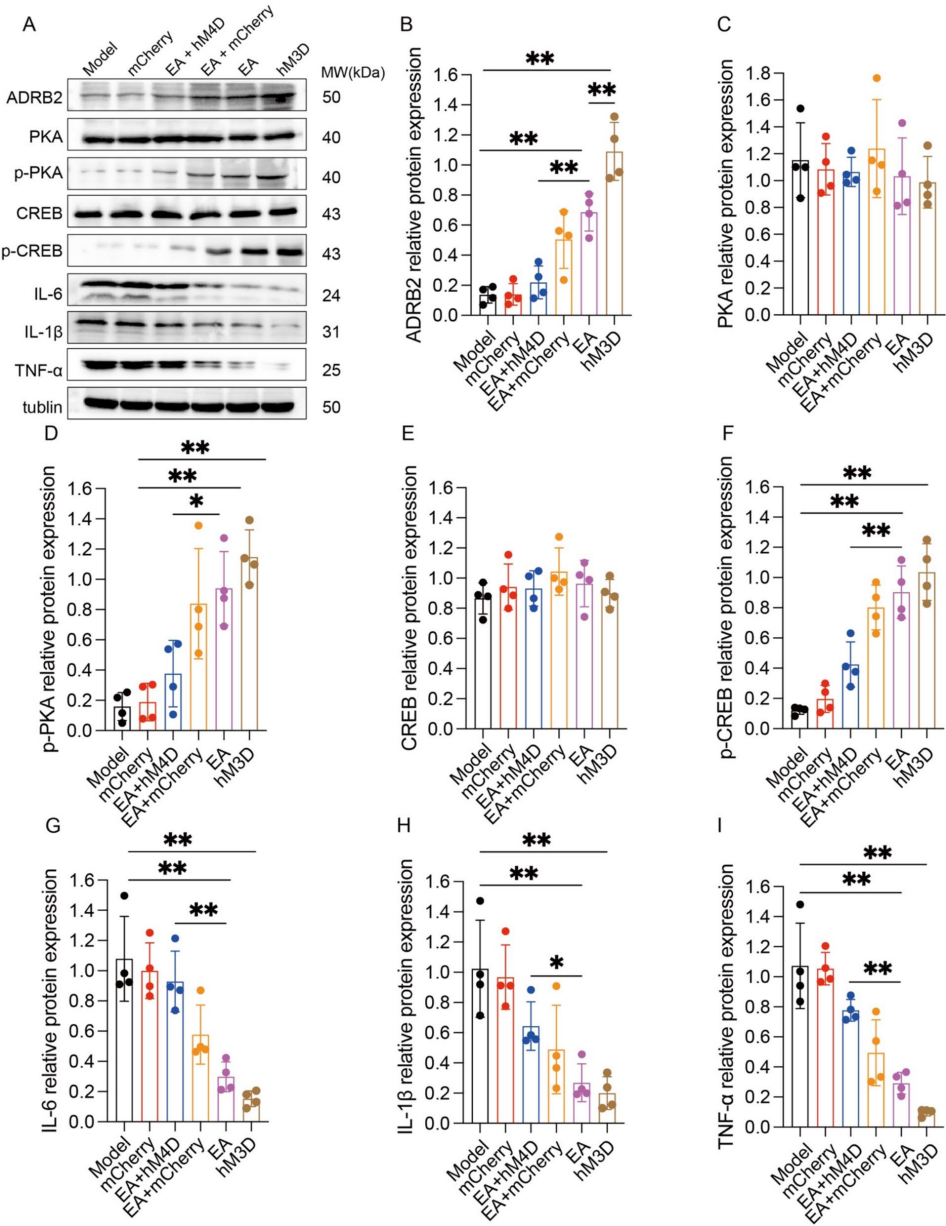
EA suppresses neuroinflammation in the hippocampus of APP/PS1 mice by activating the β2-AR/PKA/CREB pathway. **A** Representative Western blots showing the levels of ADRB, PKA, p-PKA, CREB, p-CREB, IL-6, IL-1β, and TNF-α in the hippocampi of the mice in each group. **B**–**I** Data represent the mean ± SD (n = 4). *P < 0.05 and **P < 0.01 by one-way ANOVA

Immunoblotting assays detected parallel alterations in hippocampal ADRB2, p-PKA, and p-CREB signaling proteins, demonstrating marked upregulation in both EA-treated and hM3D-activated groups relative to the model group (p < 0.01, Fig. 10B, D, F). Strikingly, EA + hM4D group induced significant downregulation of these pathway components veusus EA group (p < 0.01, Fig. 10B, D, F). Complementary neuroinflammatory profiling revealed substantial suppression of IL-6, IL-1β, and TNF-α in EA and hM3D groups relative to the model group (p < 0.01, Fig. 10G–I). Inversely, these inflammatory cytokines were significantly upregulated in the EA + hM4D group than in the EA group (p < 0.01, Fig. 10G–I). Collectively, these data delineate a mechanistic cascade whereby EA-mediated activation of the NTS-LC noradrenergic circuit potentiates β2-AR/PKA/CREB signaling pathway, consequently attenuating neuroinflammatory cascades through glial cytokine suppression.

## Discussion

**Fig. 11:**
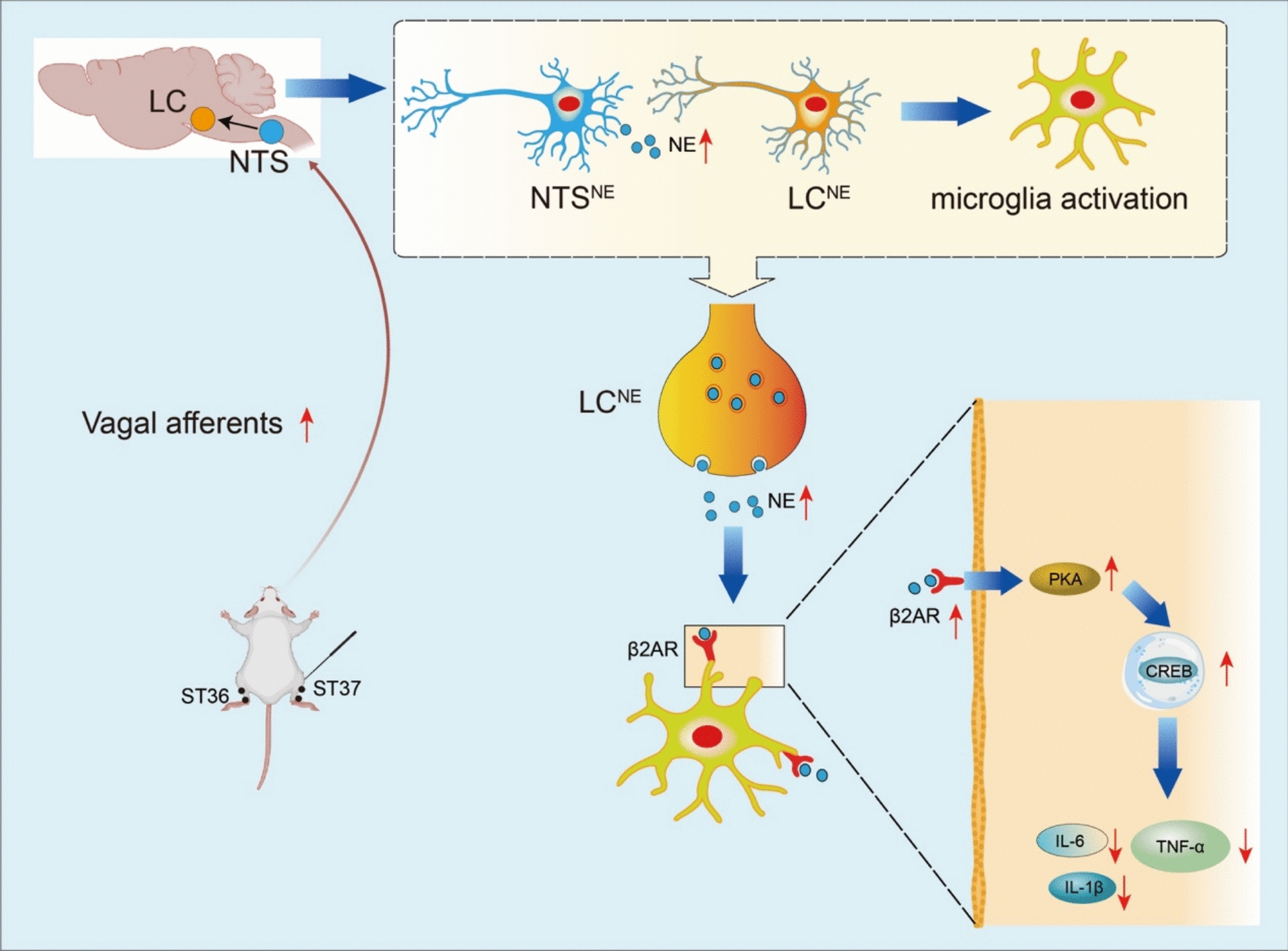
Mechanistic model illustrating that EA mitigates neuroinflammation and cognitive dysfunction in APP/PS1 mice via GVAF-dependent NTS‒LC circuit activation

The concept of gut‒brain coherence therapy, grounded in the brain‒gut axis theory, is receiving increasing attention as a potential intervention for neurological disorders such as AD, multiple sclerosis, Parkinson's disease, and depression [8]. Accumulating evidence suggests that gastrointestinal-derived signaling molecules, including 5-HT, ghrelin, and leptin, modulate memory, mood, and appetite through the gastric vagal sensory system [43, 49–51]. These findings position GVAF-mediated neural circuits as promising therapeutic targets for AD-related cognitive impairment. While previous studies have established EA as an effective modulator of vagus nerve activity for alleviating gastrointestinal dysfunction [52–54], depression [55], and sleep disorders [56], its potential role in ameliorating AD-related cognitive deficits via vagal afferents remains unexplored. In the present study, we demonstrated that EA at ST36 and ST37 activates the NTS-LC noradrenergic circuit via vagal afferents, which subsequently activates the ADRB2/PKA/CREB pathway to mitigate neuroinflammation and cognitive dysfunction in APP/PS1 mice, which has not yet been reported (Fig. 11).

Notably, vagal afferent fibers constitute approximately 80% of total vagus nerve fibers, predominantly innervating the gastrointestinal tract to relay visceral signals to the NTS [57]. These projections further integrate with higher brain regions to influence mood, appetite, memory, and central immune responses [58–60]. In contrast, efferent vagal fibers (20% of total fibers) primarily mediate top-down gastrointestinal modulation [61]. Intriguingly, GVAF activation has been linked to dopaminergic neuron stimulation in the substantia nigra and ghrelin receptor-mediated enhancement of hippocampus-dependent contextual memory [62]. Our experimental blockade of vagal afferent neurons via CCK-SAP injection into the nodose ganglion abolished the benefits induced by EA, directly implicating GVAF signaling in the improvement of EA-mediated AD. In addition, it has been reported that gut microbiota metabolites can directly regulate brain activity through vagal afferent pathways [10, 63, 64]. Although the present study did not investigate alterations in the gut microbiota, EA might enhance the anti-inflammatory effects of the NTS-LC pathway by potentially improving gastrointestinal motility and intestinal barrier function. These improvements could indirectly increase SCFA production, thereby synergistically amplifying therapeutic outcomes [65, 66]. Future investigations should integrate metagenomics and metabolomics approaches to elucidate the multilevel regulatory mechanisms of EA on the microbiota-vagal nerve-brain axis.

The NTS, as the primary relay hub for vagal afferent input, appears critical for transmitting peripheral EA stimulation to the brain [41, 67, 68]. This finding aligns with previous observations that GVAF inhibition suppresses anxiety-like behaviors in colitis models by disrupting the NTS-LC-amygdala circuit [44]. Our findings extend this paradigm by showing that GVAF blockade reduces neuronal excitability in both the NTS and the LC, concomitant with diminished LC norepinephrine synthesis. Given the critical role of the LC as the central noradrenergic hub—with widespread projections to cognition-associated regions such as the hippocampus, prefrontal cortex, and insular cortex—this circuit likely modulates AD pathogenesis through NE-dependent mechanisms. The LC-NE system is implicated in AD-related neuroinflammation, blood‒brain barrier dysfunction, and tau pathology, further underscoring its therapeutic relevance [69–71]. Our neural tracing experiments corroborated the existence of NTS-LC projections, supporting their functional significance.

Mechanistically, vagus nerve stimulation enhances hippocampal and cortical NE levels by increasing DBH in LC neurons, thereby potentiating synaptic transmission in hippocampal circuits. β-Adrenergic receptor antagonists effectively block this effect. Notably, EA can ameliorate cognitive deficits in a mouse model of vascular dementia through hippocampal NE elevation and β1-AR activation [72], which mirrors our observation that EA at ST36 and ST37 rescues neuroinflammation via NTS-LC-driven NE upregulation. The LC-NE-microglia axis has emerged as a pivotal mediator of neuroinflammation in AD [73, 74]. It has been demonstrated that the activation of LC-spinal cord descending noradrenergic pathways suppresses microglial proinflammatory responses [75]. Additionally, β2-AR agonists induce conversion from an M1-like phenotype to an M2-like phenotype in LPS-activated microglia via the activation of cAMP/PKA/CREB signaling [48]. Li et al. reported that activation of the cAMP/PKA/CREB signaling pathway is involved in EA-induced cognitive improvement in cerebral multi-infarction rats [76]. This finding aligns with recent work showing that low-intensity EA at ST36 activates vagus nerve-adrenal anti-inflammatory pathways [26]. Our findings establish that GVAF-dependent NTS‒LC circuit activation underpins the dual modulatory effects of EA on neuroinflammation and synaptic integrity in AD models.

The preservation of synaptic ultrastructure observed through spine morphological analysis is further substantiated at the molecular level by considering the essential roles of key synaptic markers. Postsynaptic density protein-95 (PSD-95) serves as a critical scaffolding organizer at excitatory synapses, anchoring NMDA/AMPA receptors and directly modulating LTP. Its expression levels strongly correlate with dendritic spine maturation and synaptic strength [77]. Complementarily, synaptophysin (SYN), as the predominant presynaptic vesicle membrane protein, functions as a quantifiable indicator of synaptic vesicle pool size and neurotransmitter release probability [4]. Our current demonstration of EA-induced PKA/CREB pathway activation offers a mechanistic framework for understanding synaptic protein regulation. CREB phosphorylation at Ser133 directly transactivates the Syp gene encoding SYN [78, 79], while simultaneously promoting PSD-95 membrane trafficking through downstream effectors like PKCζ [80]. This aligns with our prior work confirming EA's specific upregulation of hippocampal PSD-95/SYN expression [36, 81], and is further corroborated by independent studies showing EA rescues SYN/PSD-95 deficits in AD models via CREB-dependent transcription [82, 83]. The consistency between our observed preservation of synaptic ultrastructure, established CREB activation, and documented EA effects on these molecular determinants triangulates robust evidence that GVAF-NTS-LC circuit activation culminates in functionally coherent synaptic restoration.

While our study provides novel insights into GVAF-mediated mechanisms of EA in AD models, several limitations warrant consideration. First, the exclusive use of male APP/PS1 mice introduces potential sex-specific biases, as estrogen has been shown to modulate both vagal tone and neuroinflammation in neurodegenerative diseases [84–87]. Future studies should include female cohorts to evaluate sex-dependent therapeutic efficacy. The absence of direct evidence for microglial polarization states and astrocytic reactivity represents a key constraint. Future investigations should employ multi-parametric flow cytometry (e.g., CD11b microglia stained for CD86/M1 and CD206/M2 markers) and astrocytic activation (e.g., GFAP/C3 colocalization) to dissect EA-induced glial phenotypic shifts. Second, although CCK-SAP-induced vagal afferent ablation effectively validated the necessity of GVAF signaling, this approach may cause nonspecific damage to nodose ganglion neurons expressing CCK receptors, potentially confounding interpretation [37]. Conditional genetic silencing of specific vagal afferent subtypes could refine mechanistic insights. Third, the therapeutic parameters of EA—such as stimulation frequency, intensity, and duration—were optimized on the basis of prior gastrointestinal studies [88] but may not represent the ideal regimen for AD-specific neuroprotection. Comparative studies systematically varying EA parameters are needed to establish dose‒response relationships. Additionally, while we focused on the NTS-LC-NE axis, other parallel pathways—such as vagal efferent modulation of gut-derived metabolites (e.g., short-chain fatty acids) or interactions with the hypothalamic‒pituitary‒adrenal axis—might contribute synergistically to the effects of EA [89–91]. Moreover, the short-term intervention window (4 weeks) in this study leaves the long-term sustainability of EA benefits unclear. Chronic AD models with extended EA treatment could reveal potential adaptive mechanisms or tachyphylaxis. Finally, translational challenges remain: the efficacy of EA in humans may vary owing to interindividual differences in vagus nerve anatomy, gut microbiota composition, or AD heterogeneity. Combining EA with biomarkers (e.g., plasma NE levels or neuroimaging of LC integrity) could help stratify patient populations likely to benefit from this approach [92].

## Conclusion

In summary, this study provides the first experimental evidence that EA at ST36 and ST37 alleviates AD-related cognitive deficits and neuroinflammation through vagal afferent-mediated activation of the NTS-LC noradrenergic pathway. By demonstrating that GVAF signaling drives the ADRB2/PKA/CREB cascade to increase synaptic plasticity and suppress microglial activation, our findings bridge the gap between peripheral neuromodulation (EA) and central neurodegenerative pathology. While limitations such as sex-specific effects, nonspecific CCK-SAP ablation, and unresolved long-term efficacy warrant further investigation, this work establishes the gut–brain vagal axis as a novel therapeutic target for AD. Future studies should optimize EA protocols, explore synergistic mechanisms with gut-derived metabolites, and validate translational potential through clinical trials integrating neuroimaging and biomarker profiling. Collectively, these results advance our understanding of how EA neuromodulation may harness endogenous neuroprotective pathways to combat AD progression.

## Data Availability

All the data supporting the findings of this study are available from the corresponding author upon reasonable request.

## Abbreviations

LC-NE: Locus coeruleus-norepinephrine
AD: Alzheimer's disease
EA: Electroacupuncture
NTS: Nucleus tractus solitarius
GVAF: Gastrointestinal vagal afferent fiber
BDNF: Brain-derived neurotrophic factor
AAVs: Adeno-associated viruses
CCK-SAP: Cholecystokinin-saporin
NOR: Novel object recognition
MWM: Morris water maze
ADRB2: Beta 2-adrenergic receptor
PKA: Protein kinase A
CREB: CAMP-response element-binding protein
CCK-AR: Cholecystokinin A receptor
